# Unilateral Pulmonary Fibrosis Secondary to Unilateral Absence of the Pulmonary Artery With Concomitant Lung Cancer: A Case Report

**DOI:** 10.7759/cureus.100242

**Published:** 2025-12-28

**Authors:** Natalia Moguillansky, Patik P Patel, Kristianna Fredenburg, Diego Moguillansky

**Affiliations:** 1 Department of Medicine, University of Florida Health, Gainesville, USA; 2 Department of Radiology, University of Florida, Gainesville, USA; 3 Department of Pathology, University of Florida College of Medicine, Gainesville, USA; 4 Department of Internal Medicine, University of Florida Health, Gainesville, USA

**Keywords:** ct chest, lung cancer, transthoracic echocardiogram, unilateral absence of the pulmonary artery, unilateral pulmonary fibrosis

## Abstract

The finding of unilateral pulmonary fibrosis secondary to unilateral absence of the pulmonary artery is a rare condition. The association with lung cancer, both ipsilateral or contralateral, is ever rarer. Unilateral pulmonary fibrosis can be caused by proximal interruption of the pulmonary artery (congenital absence of pulmonary artery), among other causes. Diagnostic modalities for the diagnosis of unilateral absence of pulmonary artery include chest X-ray, computed tomography, echocardiogram, MRI, and aortogram, with contrasted CT chest being the gold standard. Clinical presentations are variable. Treatment includes the treatment of recurrent infections, hemoptysis, pulmonary hypertension, and possibly re-anastomosis. We present the case of a 69-year-old man with unilateral (left-sided) pulmonary fibrosis secondary to unilateral absence of the left pulmonary artery with concomitant lung cancer. This case highlights the association between unilateral pulmonary fibrosis caused by the absence of a unilateral pulmonary artery and the occurrence of lung cancer.

## Introduction

The presence of unilateral pulmonary fibrosis is a rare lung finding. There are a few etiologies for the condition that can cause it, such as proximal interruption of the pulmonary artery, pulmonary vein thrombosis, ipsilateral single lung ventilation, selective aspiration, and, more commonly, radiation pneumonitis [1-4]. Unilateral absence of pulmonary artery (UAPA) is a rare cardiovascular anomaly that has variable clinical presentations from asymptomatic to dyspnea of exertion, frequent infections, hemoptysis, and pulmonary hypertension (PHT). The association between UAPA and lung cancer has only been published in case reports. We present the case of a 69-year-old man with left-sided pulmonary fibrosis secondary to unilateral absence of the left pulmonary artery with concomitant lung cancer.

## Case presentation

A 69-year-old man who had a history of prior coronary artery bypass surgery (CABG) and insulin-dependent diabetes presented to the outpatient pulmonary specialty clinic for evaluation of an abnormal chest computed tomography (CT). He was a lifelong nonsmoker and had symptoms of shortness of breath and productive cough present for 6-12 months. Evaluation at an outside facility had included chest CT and bronchoscopy with biopsy, which was reported as unremarkable by the patient.

Physical examination findings

On presentation, the patient was afebrile, with a heart rate of 76 beats/minute and blood pressure of 138/74 mmHg. Oxygen saturation was 97% on room air. On general inspection, he was in no acute respiratory distress and was fully alert and oriented. Chest auscultation revealed crackles in the left lower lobe. His abdominal, cardiovascular, and neurological examinations were normal.

Diagnostic studies

**Figure 1:**
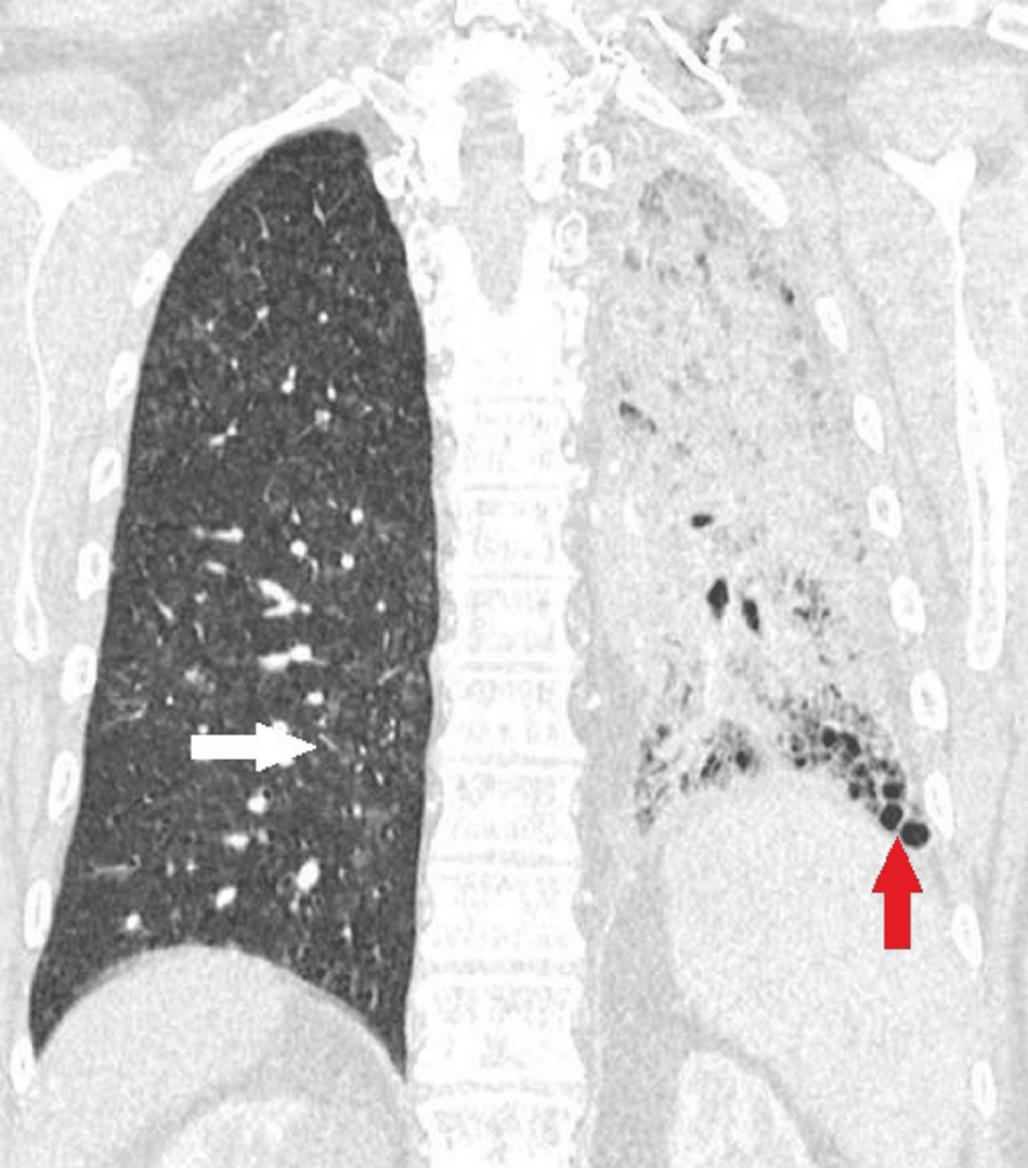
Lung window, coronal view. The CT image demonstrates decreased left lung volume relative to the right with mediastinal shift to the left. There is extensive left lung airspace consolidation with honeycombing in the left lower lobe (red arrow), as well as bronchiectasis, consistent with asymmetric pulmonary fibrosis of the left lung. On the contralateral lung (right), there are ground-glass nodules in the right middle and right lower lobes (white arrow) with small volume consolidation in the dependent right lower lobe.

**Figure 2:**
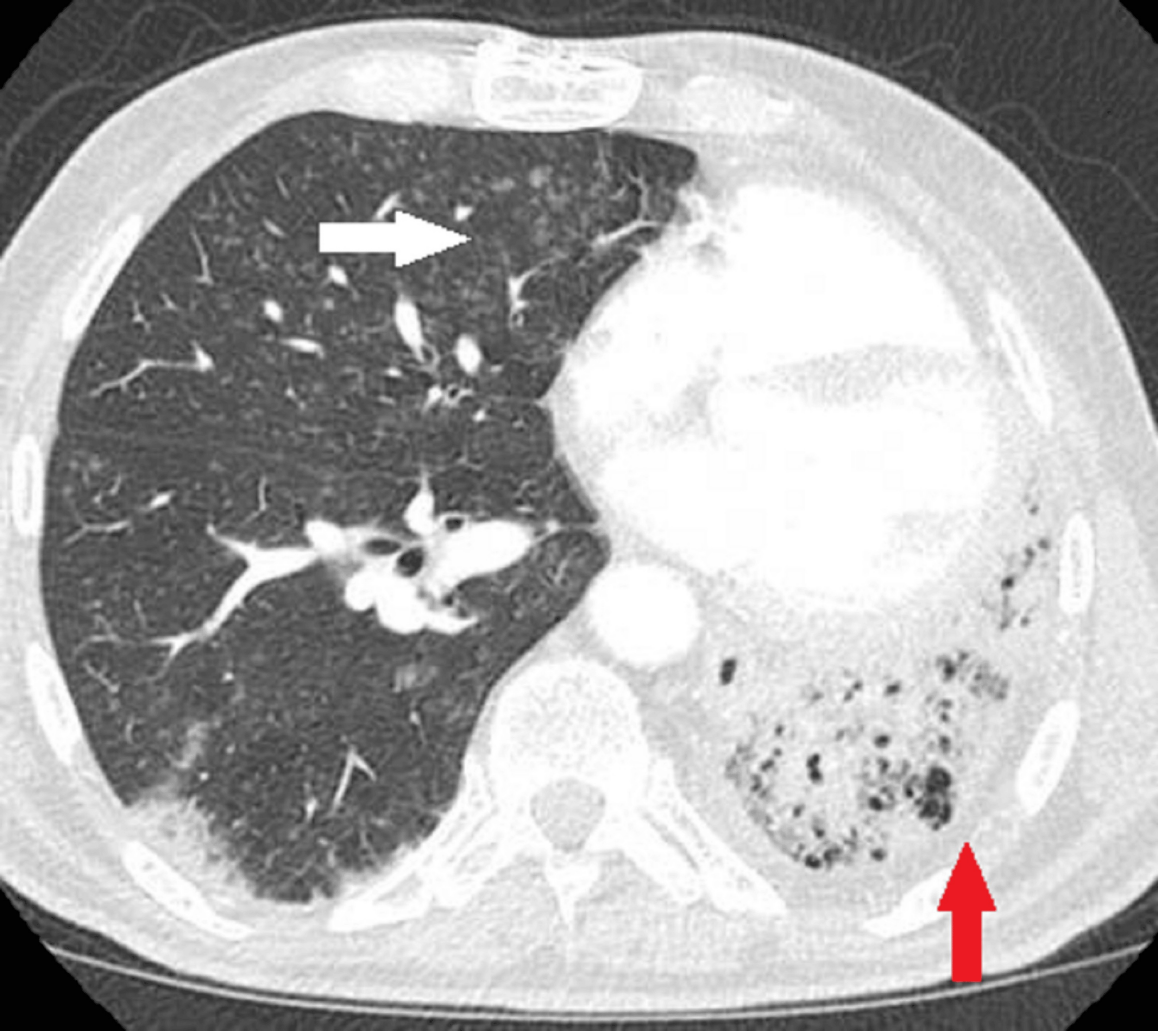
Lung window, axial view. The CT image demonstrates decreased left lung volume relative to the right with mediastinal shift to the left. There is extensive left lung airspace consolidation with honeycombing in the left lower lobe (red arrow), as well as bronchiectasis, consistent with asymmetric pulmonary fibrosis of the left lung. On the contralateral lung (right), there are ground-glass nodules in the right middle and right lower lobes (white arrow) with small volume consolidation in the dependent right lower lobe.

High-resolution CT showed severe unilateral (left) interstitial lung disease with a honey-combing pattern and superimposed left lung infiltrates, as well as left lung hypoplasia with right aortic arch (Figures 1-2).

**Figure 3:**
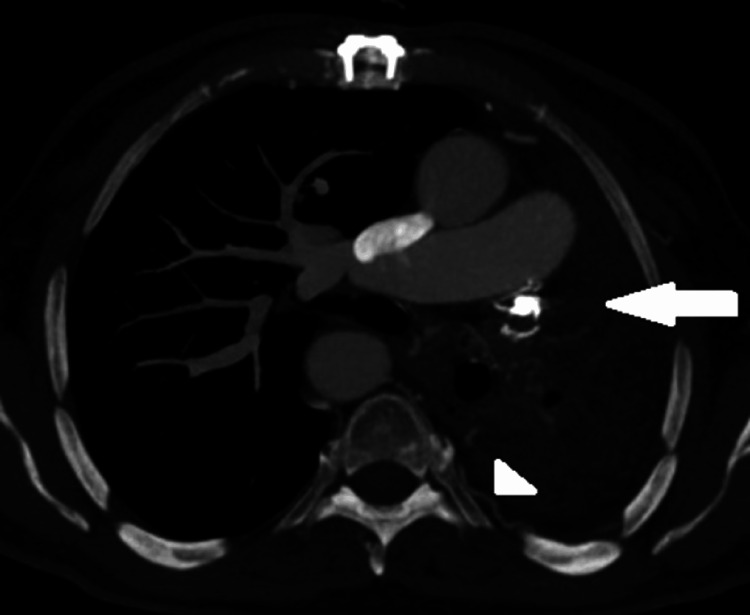
Axial soft tissue window. The CT angiographic image of the chest demonstrates unilateral absence of the left pulmonary artery (white arrow). Numerous tiny hypertrophied bronchial collateral vessels are seen within the left lung (arrowhead).

**Figure 4:**
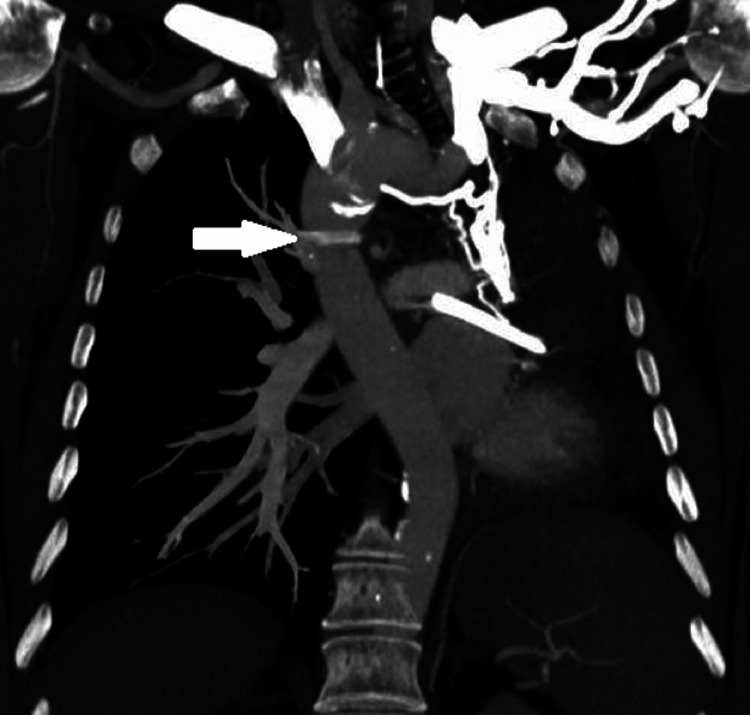
Coronal soft tissue window. Maximum intensity projection (MIP) computed tomography angiographic image demonstrates a right-sided aortic arch (arrow).

**Figure 5:**
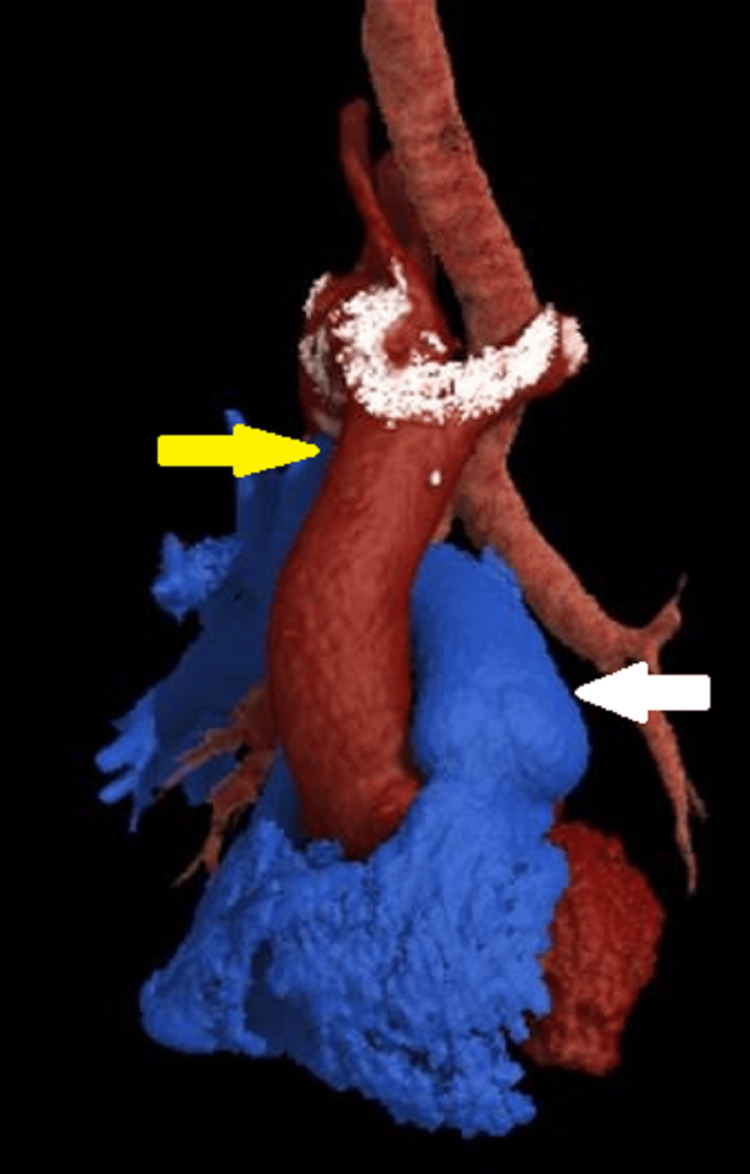
Volume-rendered three-dimensional (3D) CT angiographic image. The image depicts the vascular abnormalities, including unilateral absence of the left pulmonary artery (white arrow), a right-sided aortic arch, and its relationship with the trachea (yellow arrow).

Further testing with CT angiography (CTA) demonstrated congenital absence of the left pulmonary artery as well as right aortic arch (Figures 3-5).

**Figure 6:**
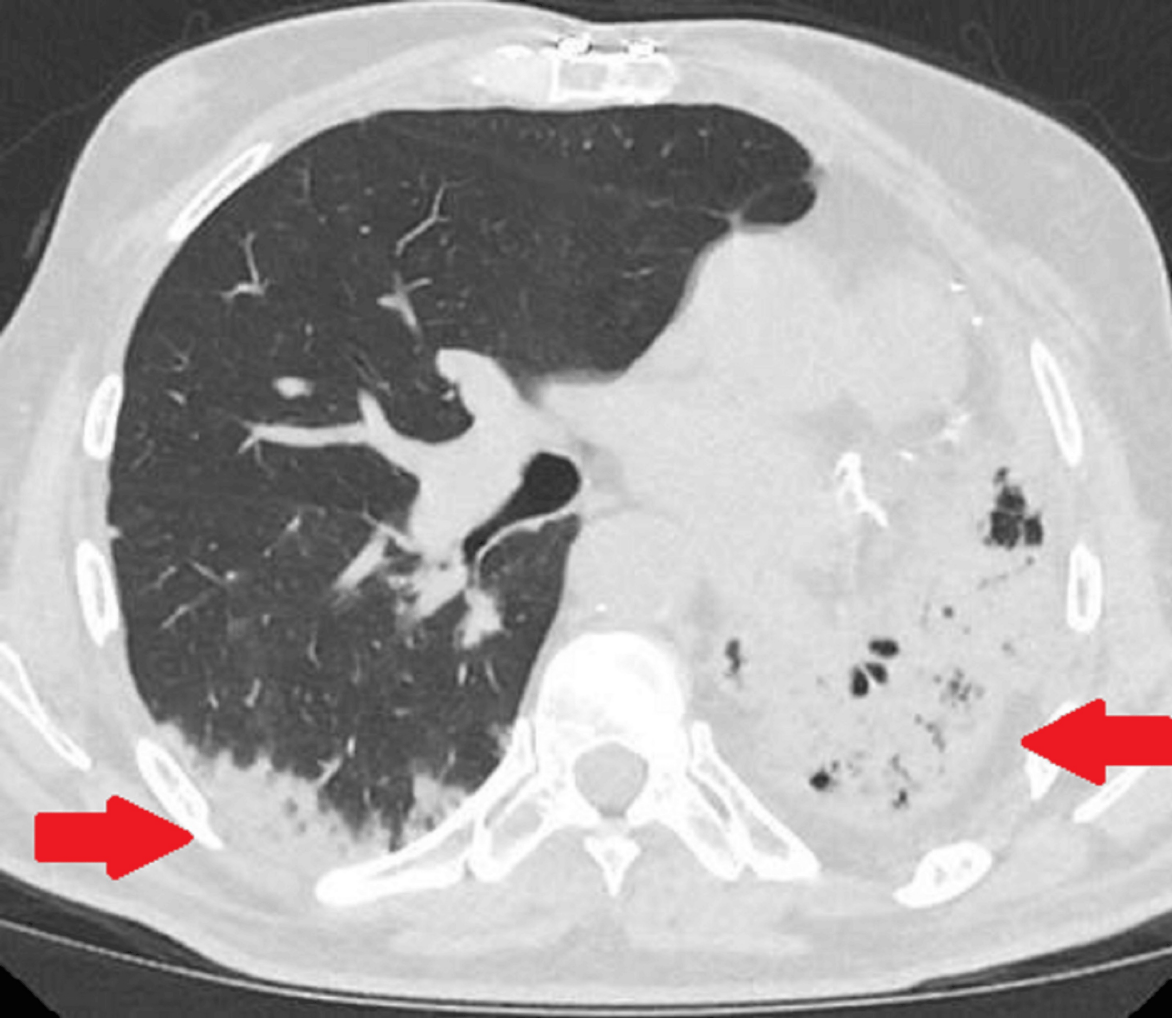
Follow-up axial non-contrast-enhanced CT lung-window image. The image obtained three months later demonstrates progressive ground-glass and solid opacities in the lower lobes, more pronounced on the left than the right, with a background of fibrosis in the left lung (red arrows).

**Figure 7:**
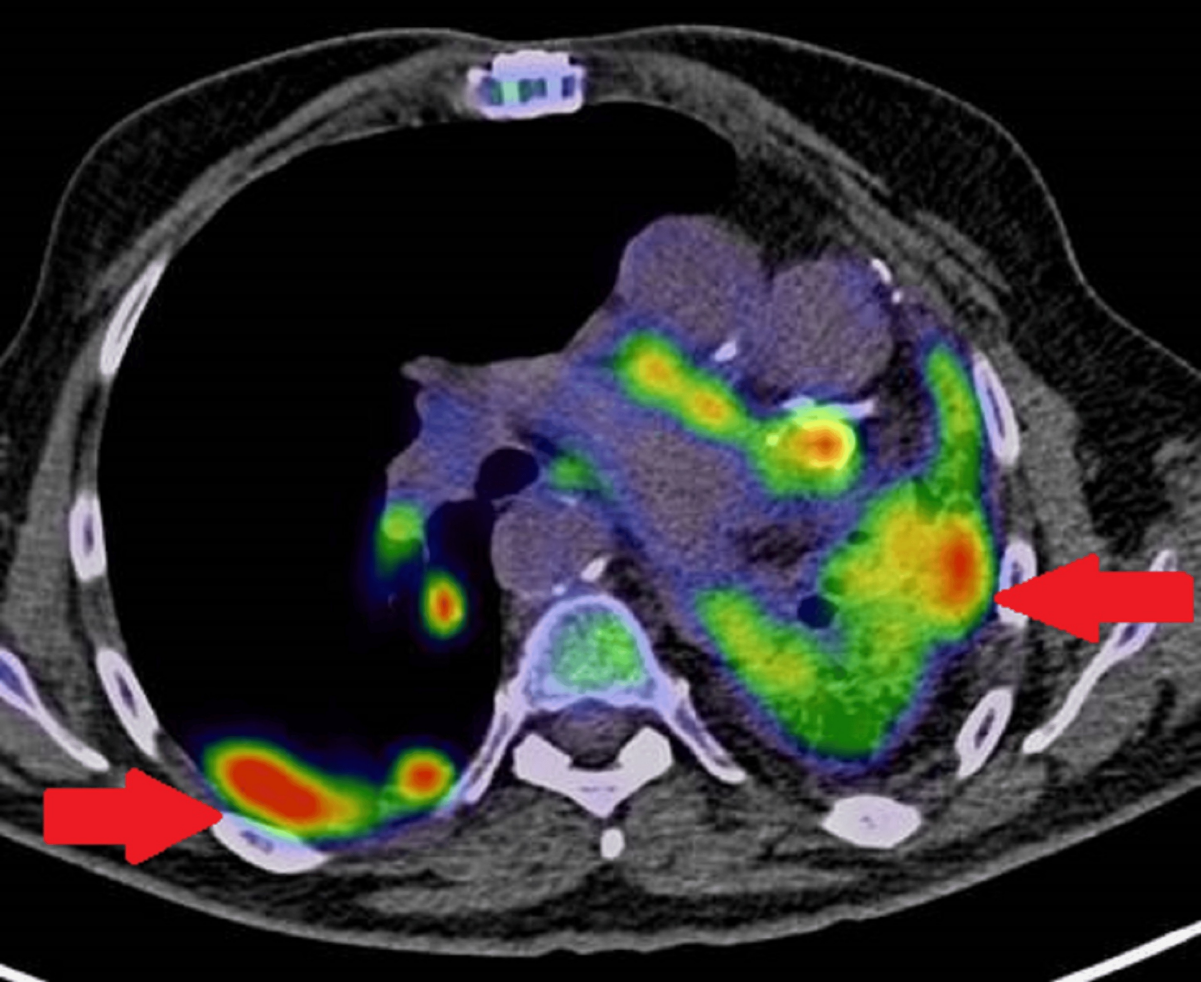
Axial positron emission tomography/computed tomography (PET/CT) image. The image demonstrates multifocal fluorodeoxyglucose (FDG) uptake in the lower lobes, greater on the left than the right (red arrows), corresponding to areas of increased ground-glass opacities noted on the diagnostic chest CT.

Due to progressive parenchymal opacities on CT (Figure 6), he eventually underwent a positron emission tomography/computed tomography (PET/CT) scan showing multifocal fluorodeoxyglucose (FDG) uptake (Figure 7).

**Figure 8:**
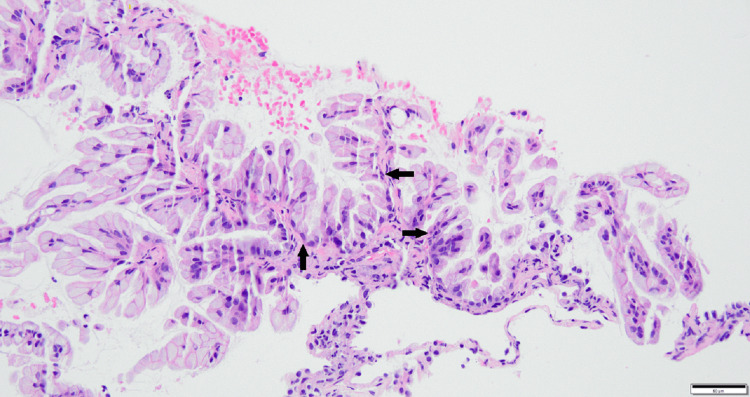
H&E-stained tissue sections (low power). Pathology shows an adenocarcinoma that has mucinous tumor cells coursing along alveolar septa. Black arrows point to alveolar septa with tumor cells.

**Figure 9:**
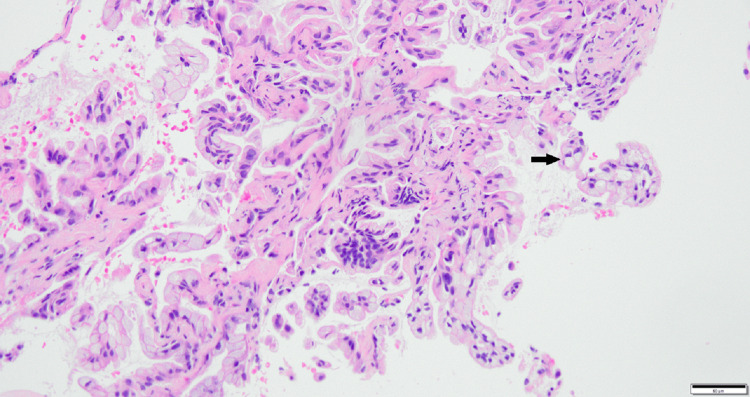
H&E-stained tissue sections (high power). Pathology shows an adenocarcinoma with tumor cells floating in pools of mucin. The black arrow points to tumor cells floating in mucin.

Transbronchial biopsies from the right lower lobe showed well-differentiated adenocarcinoma with mucinous features (Figures 8-9).

The final diagnosis was unilateral pulmonary fibrosis secondary to congenital absence of the left pulmonary artery with hypoperfusion of the left lung, complicated with lung cancer. It was determined that the patient had stage IV lung cancer without disease outside the chest. However, because he had bilateral lung cancer, he was not deemed a good candidate for radiation therapy. He was therefore offered chemotherapy with platinum/pemetrexed and was referred to a local oncology center.

For empiric treatment of cough, he was started on airway clearance with albuterol, 3% saline nebulized, and a flutter valve twice/day.

## Discussion

Unilateral pulmonary fibrosis is a very rare lung finding. It has been published in the literature in case reports and series as caused by proximal interruption of the pulmonary artery, pulmonary vein thrombosis, ipsilateral single lung ventilation, and selective aspiration [1-3,5-7]. Radiation pneumonitis is a more common cause [4].

Proximal interruption of the pulmonary artery is usually associated with UAPA. UAPA is a rare congenital vascular malformation (prevalence 1/200,000 individuals) [8] that results from a developmental abnormality involving the connection between the sixth bronchial arch artery and the pulmonary trunk. The clinical presentation of UAPA is highly variable [1]. While children born with UAPA can present with heart failure and/or PHT, many remain completely asymptomatic. The presentation in older adults can similarly range from asymptomatic to exercise intolerance, hemoptysis, PHT, and recurrent respiratory infections. UAPA can present as an isolated vascular anomaly or in association with other congenital anomalies such as Tetralogy of Fallot, atrial or ventricular septal defects, coarctation of the aorta, right aortic arch, truncus arteriosus, patent ductus arteriosus, or pulmonary atresia. UAPA can be suspected on chest radiographs, which may show ipsilateral small hemithorax, elevation of the ipsilateral hemidiaphragm, mediastinal and tracheal shift toward the affected side, absent ipsilateral hilar shadow, diminished pulmonary vascular markings, contralateral lung hyperinflation, and prominent pulmonary artery, and crowding of ribs. While the diagnosis can be suspected by echocardiogram, definitive diagnosis usually requires a chest CTA or a cardiac magnetic resonance imaging (MRI). Cross-sectional CT imaging shows an absent pulmonary artery that terminates within 1 cm of its expected origin from the main pulmonary artery. Other findings may include collateral circulation, mosaic parenchymal changes, bronchiectasis, and ipsilateral pulmonary fibrosis. CTA is the modality of choice for diagnosing UAPA as it can confirm the absence of the pulmonary artery as well as delineate the presence of any systemic collateral supply (bronchial, intercostal, subdiaphragmatic, internal mammary arteries) if present. MRI provides functional assessment of flow as well as quantification of right ventricular (RV) dimensions and systolic function, which may be useful for longitudinal follow-up. Nuclear medicine perfusion scintigraphy can also demonstrate the absence of perfusion in the affected ipsilateral lung and is sometimes used to corroborate CTA findings. An echocardiogram is a valuable tool that may be helpful for non-invasive assessment of RV size/function as well as RV/pulmonary artery pressures. Invasive cardiac catheterization can provide invasive hemodynamics as well as a pulmonary angiogram to further assess the pulmonary arteries. An aortogram can also be obtained during the catheterization to look for aorto-pulmonary collaterals that may be able to be unifocalized to the central pulmonary artery, especially in younger patients.

There are no reference guidelines regarding treatment. Asymptomatic patients without PHT or RV dysfunction can usually be followed clinically. Younger patients with multiple aorto-pulmonary collaterals to the affected lung can be considered for surgical unifocalization, where the aorto-pulmonary collaterals are re-anastomosed to the central pulmonary artery. Specific treatment for hemoptysis, pulmonary infections, and PHT should be pursued as they develop. Besides unifocalization in younger patients with significant collaterals, surgical treatment options include total pneumonectomy or closure of selected collateral arteries. Hemoptysis can be treated with embolization of the systemic collaterals or even pneumonectomy. Pharmacotherapy for lung infections and PHT can also be considered.

The association between UAPA and lung cancer is even rarer [9]. There is no evidence in the literature that cancer could be caused by lung hypoperfusion, and this association seems to be coincidental. The study by Wang et al. [10] reported nine cases published in the English Literature in 2020, with only one case published since then. Lung cancer associated with UAPA was reported to be ipsilateral in six out of 10 of the published cases [8,10-12]. Pneumonectomy seems to be a reasonable treatment for lung cancer with ipsilateral UAPA. Lung cancer in the contralateral UAPA would require limited resection, such as segmentectomy or wedge resection [12]. Preparing with an extracorporeal membranous oxygenator preoperatively would be thoughtful.

## Conclusions

Unilateral pulmonary fibrosis is a rare lung finding that can be caused by proximal interruption of the pulmonary artery, such as UAPA, which is a rare vascular malformation. The presentation can range from asymptomatic to exercise intolerance, hemoptysis, PHT, or recurrent pulmonary infections. The definitive diagnosis of UAPA can be made by chest CTA or MRI. The treatment of UAPA includes treatment of hemoptysis with embolization or lung resection, lung infections with antibiotics, and PHT with specific pulmonary arterial hypertension (PAH) pharmacotherapy. Surgical interventions include total pneumonectomy, closure of selected collateral arteries, or a primary versus staged pulmonary artery anastomosis. UAPA can rarely be associated with lung cancer. The present case highlights the occurrence of unilateral pulmonary fibrosis caused by UAPA associated with lung cancer, which is likely coincidental.
